# Framework for the Classification of Emotions in People With Visual Disabilities Through Brain Signals

**DOI:** 10.3389/fninf.2021.642766

**Published:** 2021-05-07

**Authors:** Jesús Leonardo López-Hernández, Israel González-Carrasco, José Luis López-Cuadrado, Belén Ruiz-Mezcua

**Affiliations:** Computer Science Department, Universidad Carlos III de Madrid, Madrid, Spain

**Keywords:** emotion classification algorithm, brain–computer interface, machine learning, visual disabilities, affective computing

## Abstract

Nowadays, the recognition of emotions in people with sensory disabilities still represents a challenge due to the difficulty of generalizing and modeling the set of brain signals. In recent years, the technology that has been used to study a person’s behavior and emotions based on brain signals is the brain–computer interface (BCI). Although previous works have already proposed the classification of emotions in people with sensory disabilities using machine learning techniques, a model of recognition of emotions in people with visual disabilities has not yet been evaluated. Consequently, in this work, the authors present a twofold framework focused on people with visual disabilities. Firstly, auditory stimuli have been used, and a component of acquisition and extraction of brain signals has been defined. Secondly, analysis techniques for the modeling of emotions have been developed, and machine learning models for the classification of emotions have been defined. Based on the results, the algorithm with the best performance in the validation is random forest (RF), with an accuracy of 85 and 88% in the classification for negative and positive emotions, respectively. According to the results, the framework is able to classify positive and negative emotions, but the experimentation performed also shows that the framework performance depends on the number of features in the dataset and the quality of the Electroencephalogram (EEG) signals is a determining factor.

## Introduction

The recognition of human emotions was proposed long ago as a way for the development of current computing, with the aim of designing machines that recognize emotions to improve the interaction between humans and computer systems (Picard, 2003). Besides, it represents a challenge since this could mean that computers respond in real time and in a personalized way to the affective or emotional states of a person (Kumar et al., 2016).

Emotions in a person play an important role in non-verbal communication and are essential for understanding human behavior (Liu et al., 2011). Moreover, some research related to analyzing emotional behavior and automatic recognition of emotions using machine learning techniques have generated high expectations. First, emotions have been studied from behavioral signals, named emotional signals (Ekman and Friesen, 1969) and from the analysis of body posture and movement (Ekman et al., 1991). On the other hand, various approaches and ways have been tested for the classification of emotions in people under different circumstances, like music (Vamvakousis and Ramirez, 2015), autism (El Kaliouby et al., 2006), the recognition of emotions using electrodermal activity sensors (Al Machot et al., 2019), or e-Healthcare applications (Ali et al., 2016).

Although the human emotional experience has a vital role in our lives, scientific knowledge about human emotions is still minimal (Soleymani et al., 2012). Consequently, emotions classification is a problem considered challenging since emotional states do not have precisely defined limits, and often, the perception between users differs. Therefore, research on the recognition and emotions classification is of importance in real-life applications (Anagnostopoulos et al., 2012; Zhang et al., 2020).

Through the recognition of speech and the processing of facial gestures, it has been possible to classify a person’s emotions, and commonly these approaches have given good results. However, it has been detected that people can manipulate these methods. Therefore, to achieve objectivity in the technique, the source of the emotion must not be easily manipulated (Ackermann et al., 2016). Consequently, a new reliable and objective approach is required to avoid these cases.

In recent years, through the brain–computer interface (BCI), the behavior and emotions of a person have been studied (Nijboer et al., 2008; Chaudhary et al., 2016; Pattnaik and Sarraf, 2018). These studies indicate that BCI technology offers an additional benefit as it is a method that cannot easily be manipulated by the person. Therefore, it is possible to obtain valid and accurate results by analyzing brain signals obtained using a BCI (Patil and Behele, 2018).

People with any sensory disability often do not have access to current technology. For this reason, it is necessary to develop new ways of communication and interaction between the human and the computer to give support to these people considering their disability and the degree it affects them. Therefore, the effective adaptation of a brain–machine interface to the recognition of the user’s emotional state can be beneficial to society (Picard, 2010).

Commonly, people with total or partial visual disability have difficulties completing their daily tasks (Leung et al., 2020). This is associated with dependence when carrying out daily activities, even in some cases with decreased physical activity (Rubin et al., 2001). Frequently, people with visual disabilities need to use support tools that allow them to interact with the environment around them, so they must alter their behavior according to their needs. Therefore, changing how people with visual disabilities communicate, intervene, and express themselves in their environment through the recognition of their emotions would improve their quality of life. Besides, this would positively impact their daily lives since it would put them on an equal footing in current technology access and use.

In various studies related to the classification of emotions through biological signals such as brain signals, music has been used as a source to induce human emotions. Besides, music is considered capable of evoking a series of emotions and affect people’s mood (Koelsch, 2010). However, music’s influence on emotions is often unknown due to individual preference and appreciation for music (Naser and Saha, 2021). The action of listening to music and psychological processes such as perception, attention, learning, and memory are involved. Therefore, music has been considered a useful tool to help study the human brain’s functions (Koelsch, 2012). Additionally, music can provoke strong emotional responses in listeners (Nineuil et al., 2020). Moreover, it has been shown that music is used for understanding human emotions and their underlying brain mechanisms (Banerjee et al., 2016). For these reasons, music is considered adequate to induce and study various human emotions, including positive and negative (Peretz et al., 1998). Taking into account various psychological aspects and the effects of music on emotions, music has been studied in the regulation of moods in people (Van Der Zwaag et al., 2013), the effects of music on memory (Irish et al., 2006), recognition of brain patterns while listening to music (Sakharov et al., 2005), etc.

Previously, this paper’s authors analyzed the research that proposes the classification of emotions in people with visual disabilities (López-Hernández et al., 2019). The results showed that new approaches that specifically consider people with visual disabilities and the study of their emotions are still required. Based on these results, the design of a system that classifies the affective states of people with visual disabilities was proposed by identifying a person’s emotional responses when they are auditory stimulated.

For the reasons mentioned above, this research’s main motivation is to provide an integrated framework for acquiring brain signals through a BCI, characterizing brain activity models, and defining machine learning models for the automatic classification of emotions, focused on people with visual disabilities.

This study expects to obtain new evidence on the application of BCIs, affective computing, and machine learning, oriented toward the development of communication and interaction alternatives between systems and people with visual disabilities.

Likewise, the challenges associated with this research are the analysis and evaluation of emotional behavior as well as the perception of the responses to an auditory stimulus of people with visual disabilities.

## Related Work

Next, a review of related works on applying a BCI for the recognition and classification of people’s emotions using machine learning algorithms is presented.

An EEG signal-based system for automatic recognition of emotions was proposed to examine different methods of extracting EEG features, channel locations, and frequency bands (Ackermann et al., 2016). Machine learning algorithms such as support vector machines (SVMs), random forests (RFs), and decision trees (DTs) were evaluated with pre-processed data for the analysis of emotions, based on physiological data provided during the training and testing tasks. In their results and experimental findings, the authors report that the RF algorithm behaves better in recognition of emotions from signals coming from the EEG. Likewise, they mention that although it is possible to recognize human emotions from other sources, the most reliable way is through EEG signals due to this approach’s objectivity.

Another study proposes the identification of four emotions through the analysis of EEG and the exploration of machine learning algorithms such as Multiclass SVM for the emotion classification task (Patil and Behele, 2018). The results indicate that the model obtains a 91.96% precision in the classification of emotions. Likewise, it is mentioned that an EEG is a more reliable data source for the study of emotions since the subject cannot alter the data.

An approach to the acquisition and processing of the EEG signals obtained using the Emotiv Epoc+ device and the evaluation of a neural network model for the classification of emotional states of people without disabilities reports results of 85.94, 79.69, and 78.13% for valence, excitement, and dominance, respectively (Sánchez-Reolid et al., 2018).

A model for identifying human emotions using EEG signals and Multi-Feature Input Deep Forest Model has been used as an alternative to classifying five emotions, neutral, angry, sad, happy, and pleasant (Fang et al., 2021). In this study, EEG signals from a public dataset for emotion analysis (DEAP) are used. Data processing involves dividing the EGG signals into several frequency bands, processing the power spectral density, the differential entropy of each frequency band, and the original signal as features of the model. Results show that the MFDF model achieves 19.53% more precision with the compared algorithms (RF, SVM, and KNN).

The detection of emotions from EEG signals is also studied by Ramirez and Vamvakousis (2012). The paper describes an automatic approach to emotion detection based on brain activity using the Emotiv Epoc+ headset. In this study, a group of men and women were stimulated auditorily with 12 sounds from the IADS database. During the extraction of features, alpha waves (8–12 Hz) and beta waves (12–30 Hz) were considered, using a bandpass filter and the Fourier analysis of frequency. Linear Declining Analysis (LDA) and SVM algorithms were evaluated for the two-class classification task. Finally, the results indicated that the best classification results for excitation and valence were 83.35 and 86.33%.

A new normalization method of features named stratified normalization is studied to classify emotions from EEG signals (Fdez et al., 2021). In this research, the SEED dataset is used, and the data on the effects of three independent variables (labeling method, normalization method, and feature extraction method) are recorded. This method proposes an alternative for the normalization of features to improve the precision of the recognition of emotions between people. The results indicate 91.6% in the classification of two categories (positive and negative) and 79.6% in the classification of three categories (positive, negative, and neutral).

Other research shows the analysis and evaluation of machine learning, SVM, and K-nearest neighbors (KNN) methods to classify a person’s emotions while observing a visual stimulus (Mehmood and Lee, 2015). In this research, five people (without disabilities) participated in the experiment, and the EEG data were recorded through the Emotiv Epoc+ headset. The processing of the EEG signals was through the EEGLAB toolbox applying the Independent Component Analysis (ICA) technique. The best result of the application of the automatic learning methods for the classification of emotions was 61% accuracy for KNN, as opposed to SVM, which obtained 38.9% accuracy.

K-nearest neighbors algorithm and its functioning to classify emotions are described by Kaundanya et al. (2015). This proposal is a method for EEG signal acquisition tasks, pre-processing, feature extraction, and emotion classification. Several subjects were stimulated for the emotions of sadness–happiness, and the data acquisition was performed with the ADInstruments device. The recorded EEG signals were processed by applying a bandpass filter (3–35 Hz) to remove the signals’ noise. The results indicated that the KNN algorithm is viable for the classification task.

Decision tree classifiers for EEG signals have been used in different research works. A fast and accurate DT structure-based classification method is used for classifying EEG data with computer cursor up/down/right/left movement images (Aydemir and Kayikcioglu, 2014). The detect epileptic seizure in EEG signals uses a hybrid system based on DT classifier and fast Fourier transform (FFT) (Polat and Güneş, 2007). DT and a BCI have also been used to assist patients who are nearly or entirely “locked-in,” i.e., cognitively intact but unable to move or communicate (Kennedy and Adams, 2003).

In summary, several approaches have been proposed to classify emotions, from voice recognition or facial expressions to BCI, to extract brain signals (EEG). Although these methods have been tested in different settings and their results are correct, the literature mentions that the most reliable method is the use of brain signals (EEG) due to its objectivity in reading the data. Additionally, the classification of emotions for the development of new systems that respond to the emotional states of a person has been analyzed in different research works. In addition, machine learning models, previously labeled datasets and different scenarios have been explored, and the results demonstrate the viability of the proposals. However, the authors consider that new scenarios must be evaluated, considering the classification of affective states in people with disabilities.

The remainder of this article is organized as follows: In section “Related Work,” the related works are discussed, and section “Materials and Methods” presents the methods and materials used in this research. In section “Proposed Framework,” the proposed framework is described, highlighting its principal features. Section “Results” shows the results obtained from the experimentation after the implementation of the proposed model. Possible causes of the results are discussed in section “Discussion.” Finally, section “Conclusion” presents the conclusions and future works related to the implementation of systems capable of recognizing and responding in real time to the emotions of a person with disabilities.

## Materials and Methods

This section describes the tools and methods used during the experimental phase of this research.

### Headset Emotiv Epoc+

**FIGURE 1:**
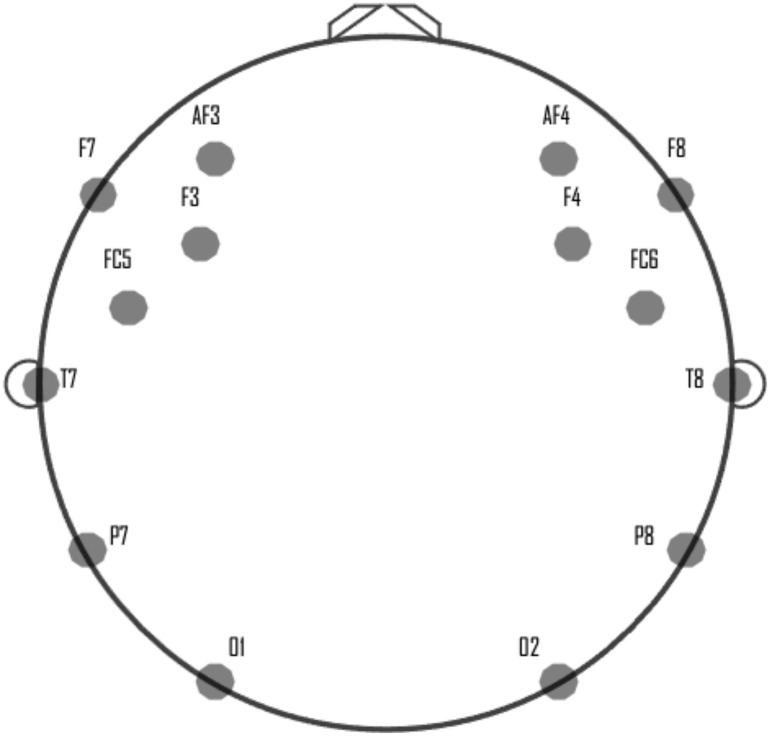
Location of the electrodes in Emotiv Epoc+ headset.

Emotiv Epoc+ is a high-resolution portable EEG device, which is used to record brain signals. This device has 14 electrodes for reading brain signals and two CML/DRL reference electrodes. It is designed to operate quickly during the tasks of acquisition and processing of brain signals (Emotiv Epoc, 2019). The configuration of the device Emotiv Epoc+, for the acquisition of the EEG signals, is supported by the sensors: AF3, F7, F3, FC5, T7, P7, O1, O2, P8, T8, FC6, F4, F8, and AF4. Figure 1 shows the electrode locations for Emotiv Epoc+.

### Participants

The present study was developed in collaboration with the ONCE social group from Madrid, Spain. This group involves the National Organization of the Spanish Blind (ONCE) and other related entities. ONCE supports people with needs derived from blindness or a severe visual impairment by providing of specialized Social Services.

This study involved seven participants, five men and two women, from whom the ONCE social group invited four people to participate in the study. The age of the participants is between 40 and 55 years old. Previously, all participants reported having normal hearing, and before the experiment, they gave their consent in a confidentiality document to process personal data and participate in the study. Likewise, they were informed of the procedure and of their right to suspend the study. Considering the participants, the experiment was carried out following the principles of the Declaration of Helsinki.

### Stimuli

For the experimentation of this study, two classical music audios with different musical styles were selected, the first being joy–happiness and the second being fear–suspense. From these, 40 stimuli (audios) with 5 s each have been generated and selected. The purpose of using stimuli of different musical styles is to induce different affective states (emotions) in the participants.

## Proposed Framework

The related work has exposed different research works for the identification of a person’s emotional responses when they are auditory stimulated. As it has been stated, despite the number of works in this area, future research is needed for developing high-performance BCI systems to allow people with needs to perform activities of daily living (Yuan and He, 2014).

Furthermore, due to the advancement of computational tools, the task of recognition and classification of human emotions based on machine learning models has generated interest (Asghar et al., 2019). For this reason, in this research, different machine learning models are evaluated looking for the one with the best performance in this problem.

This manuscript presents a new framework focused on people with visual disabilities, taking into account those findings. The framework is composed of different components and stages:

(A) Data acquisition: EEG signals data acquisition by a BCI interface and brain activity models characterization.
(B) Pre-processing: Analysis of brain activity models and EEG data signals for feeding the training and test process of the machine learning models.
(C) Machine Learning: Definition and evaluation of different machine learning models.
(D) Classification: Automatic classification of basic emotions (positive or negative).

**FIGURE 2:**
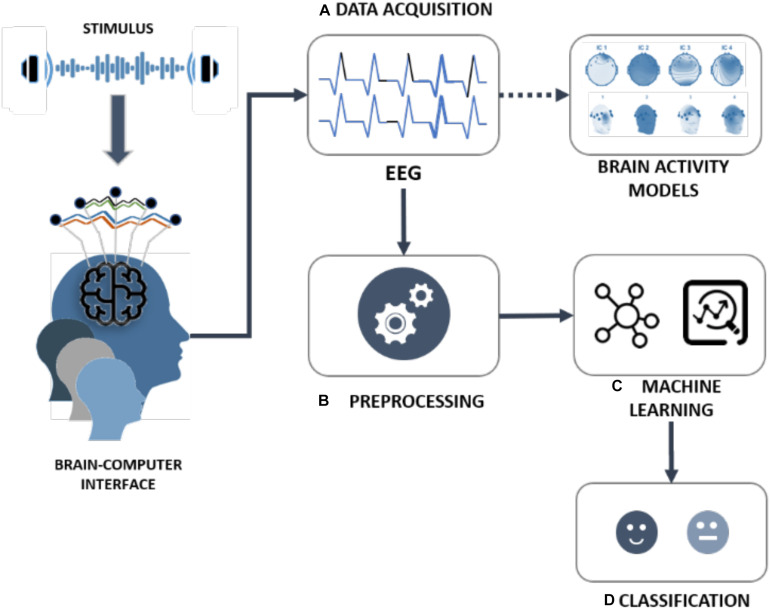
Framework for the classification of emotions in people with visual disability.

The different components and stages of the framework are shown in Figure 2 and detailed in the following subsections.

### Data Acquisition

The first step of the proposed framework is data acquisition and collection. During the presentation of a stimulus, the Emotiv Epoc+ device is used to read and record real-time people’s brain activity. These sensors are placed on the participants as shown in Figure 1, following the guidelines of the international 10–20 standard for electrode positioning (Sharbrough et al., 1991).

#### Experiments

Before starting the experimentation stage, the participant is informed of the data recording procedure and the process to evaluate each stimulus. Subsequently, the participant performs a test with the Emotiv Epoc+ headband to ensure the correct reading of the data; in addition, volume tests with the audio device were carried out to validate that the participant is comfortable.

During each test, the task of inducing different affective states or emotions in the participants was presented using auditory stimulation. Each participant listens to 40 previously selected auditory stimuli, divided into four groups of 10 stimuli, where each stimulus is presented for 5 s. Between each stimulus, the participant has 3 s to rate the stimulus heard and 5 s of silence to evoke a neutral emotional state in the participant. The experimentation process is divided into four stages that are described below.

##### Experiment 1

In this test, 10 trials’ data are recorded presenting 10 stimuli (in that order): five of Joy–Happiness and five of Fear–Suspense. Likewise, the participant will rate each stimulus as positive (pleasant) or negative (unpleasant) according to their musical preferences.

##### Experiment 2

During this test, 10 trials are carried out from presenting 10 stimuli (in random order): five of joy–happiness and five of fear–suspense. In this test, the participant rates each stimulus according to their musical preferences, positive (pleasant), or negative (unpleasant), respectively.

##### Experiment 2

For this test, data from 10 trials are saved by an orderly presentation of five joy–happiness stimuli and five fear–suspense stimuli. Each participant rates each stimulus according to their musical preferences (positive or negative).

##### Experiment 4

In this last test, a random order is considered for recording the data of 10 trials using five stimuli of joy–happiness and five of fear–suspense, respectively. Each stimulus is rated positive or negative, depending on each participant.

Finally, the data obtained from the signal’s EEG are digitized into a file for each user. The file contains all the information related to the experiment: type of stimulus, the time interval the stimulus was presented, the wave magnitude for each electrode, and the participant’s evaluation for each stimulus.

### Pre-processing

This component of the framework processes the source data of the EEG signals of each participant obtained in the data acquisition stage. A component of the framework extracts the signal from each sensor, applies FFT, and filters the signal using a filter band pass between 0.5 and 30 Hz. The result of this task is the conversion of the data into the signal frequencies, delta (0.5–4 Hz), theta (4–8 Hz), alpha (8–15 Hz), beta (15–30 Hz), and an average wave magnitude of each electrode, reducing the amount of data generated and therefore improving its understanding.

### Machine Learning

The machine learning component of the framework includes eight machine learning classifiers: RF, logistic regression (LR), multilayer perceptron (MLP), KNN, Linear Discriminant Analysis (LDA), Naive Bayes (NB), DT, and neural networks (NNs) where different experiments are configured, altering the main parameter for each algorithm.

The KNN method is a popular classification method in data mining and statistics because of its simple implementation and significant classification performance. KNN classifier is a type of instance-based learning or non-generalizing learning: it does not attempt to construct a general internal model but simply stores instances of the training data. Classification is computed from a simple majority vote of the nearest neighbors of each point: a query point is assigned to the data class, which has the most representatives within the nearest neighbors of the point. However, it is impractical for traditional KNN methods to assign a fixed *k* value (even though set by experts) to all test samples (Zhang et al., 2017, 2018). Considering this, for the KNN classifier, the number of neighbors (n_neighbors) parameter is modified in the experimentation (moving from 1 to 20).

Random forest classifier is an ensemble classifier that produces multiple DTs, using a randomly selected subset of training samples and variables. Over the last two decades, the use of the RF classifier has received increasing attention due to the excellent classification results obtained and the speed of processing (Belgiu and Drǎgu, 2016). RF algorithm is a meta estimator that fits a number of DT classifiers on various subsamples of the dataset and uses averaging to improve the predictive accuracy and control over-fitting. The subsample size is always the same as the original input sample size, but the samples are drawn with replacement in this case. The number of trees (NT) in the RF algorithm for supervised learning has to be set by the user. It is unclear whether the NT parameter should be set to the largest computationally manageable value or whether a smaller NT parameter may be enough or, in some cases, even better (Cutler et al., 2012; Probst and Boulesteix, 2018). RF is an algorithm that has been shown to have excellent performance for classification tasks. It uses a set of trees (n_estimators), which are based on the technique of sampling the data (Vaid et al., 2015). Taking this into account, for the RF classifier, the NT parameter (n_estimators) is modified in the experimentation (moving from 1 to 25).

Decision trees are a non-parametric supervised learning method used for classification and regression. The goal is to create a model that predicts the value of a target variable by learning simple decision rules inferred from the data features. Many systems have been developed for constructing DTs from collections of examples (Quinlan, 1987). The study for the number of features to consider when looking for the best split has been under research for many years (Kotsiantis, 2013; Fratello and Tagliaferri, 2018). Taking this into account, for the DT classifier, the number of features (max_features) for the best split is modified in the experimentation (moving from 1 to 14).

Linear Declining Analysis algorithm is a classifier that works with a linear decision limit. However, it is also considered a technique for feature extraction and dimensionality reduction. LDA projects the data in a vector space with a covariance matrix and an average vector of lower dimensions. Finally, the samples are classified according to the closest average vector (Torkkola, 2001; Ye et al., 2005). Furthermore, LDA has been used to reduce the number of dimensions in datasets, while trying to retain as much information as possible.

Logistic regression is a statistical method that examines the relationship between a dependent variable (target) and a set of independent variables (input), which is applied in regression problems, binary classification, and multiclassification. The LR algorithm technique is based on finding a prediction function and a loss function and identifying the parameters that minimize the loss function. In LR classification problems, you first create a cost function and then apply an iterative optimization process to identify the optimal model parameters (Tsangaratos and Ilia, 2016; Fan et al., 2020).

Naive Bayes classifier is a probabilistic algorithm known for being simple and efficient in classification tasks. From a set of training data, it estimates a joint probability between the features (X) and the targets (Y) (Tsangaratos and Ilia, 2016). NB learns the parameters separately for each attribute, which simplifies learning, even in large datasets (Mccallum and Nigam, 1998).

Multilayer perceptron is a type of NN frequently used in pattern recognition problems. Due to its ease of implementation and adaptability to small datasets (Subasi, 2007). The MLP implementation consists of three sequential layers: input (s), hidden (s), and output (s). The hidden layer processes and serves as an intermediary between the input and the output layer (Orhan et al., 2011).

A NN is a model that has been studied in supervised learning approaches. These models are composed of a large number of interconnected neurons, on which parallel calculations are performed to process data and obtain certain knowledge. The learning of a NN is based on rules that simulate biological learning mechanisms. For classification tasks, NN models are important for their ability to adapt and fit the data (Subasi, 2007; Naraei et al., 2017).

Taking this into account, Table 1 shows the configuration of each algorithm and the parameters that have been modified during experimentation.

**TABLE 1:** Classifiers configuration.

*Algorithm*	*Parameter*	*Value*	*Comment*
K-nearest neighbors (KNN)	Algorithm	“Auto”	KNN will attempt to decide the most appropriate algorithm based on the values passed to fit method: Ball tree, K-d tree or brute-force search
	Leaf_size	10 to 30	Leaf size passed to BallTree or KDTree algorithms
	Metric		“Minkowski”
	n_neighbors	From 1 to 20	Number of neighbors to use by default for queries
	P	2	Power parameter for the Minkowski metric. When *p* = 2, metric is Euclidean_distance
Random forest (RF)	Max_depth	None	The maximum depth of the tree
	Max_features	“Auto”	The number of features to consider when looking for the best split
	Max_leaf_nodes	Unlimited	Grow trees in best-first fashion. Best nodes are defined as relative reduction in impurity
	Min_impurity_split	1e^–7^	Threshold for early stopping in tree growth.
	n_estimators	From 1 to 1000	The number of trees in the forest
Decision tree (DT)	Max_features	From 1 to 56	The number of features to consider when looking for the best split
	Max_depth	None	The maximum depth of the tree. If none, then nodes are expanded until all leaves are pure or until all leaves contain less than min_samples_split samples
	Min_samples_split	From 2 to 5	The minimum number of samples required to split an internal node
	Splitter	best	It defines the strategy to choose the split at each node
Linear Discriminant Analysis (LDA)	Solver	Svd, lsqr, and eigen	Solver that will use the algorithm
	Tol	0.0001, 0.001, and 0.01	Threshold for Solver Range Estimation (SVD)
	n_components	None	Number of components, for dimensionality reduction
	Store_covariance	False	Allows to calculate the class covariance matrix
Logistic regression (LR)	Max_iter	From 100 to 2,000	Maximum number of iterations taken for the solvers to converge
	Solver	Liblinear, lbfgs	Algorithm to use in the optimization problem.
	Multi_class	Ovr, auto	“ovr,” for a binary problem
	Tol	1e-4, 1e-3, and 1e-2	Tolerance for stopping criteria
	Penalty	L1 and L2	Used to specify the norm used in the penalization
Naive Bayes (NB)	Priors		Prior probabilities of the classes
	Var_smoothing	1e-9, 1e-7, and 1e-5	Portion of the largest variance of all features that is added to variances for calculation stability
Multilayer perceptron (MLP)	Hidden_layer_sizes	From 28 to 56	The *i*th element represents the number of neurons in the ith hidden layer
	Max_iter	5,000	Maximum number of iterations
	Early_stopping	True	Whether to use early stopping to terminate training when validation score is not improving
	Activation	Relu	Activation function for the hidden layer
	Alpha	0.0001 and 0.001	L2 penalty (regularization term) parameter
Neural network (NN)	Input_dim	From 28 to 56	Number of neurons in the input layer
	Kernel_initializer	Uniform	Initializers define the way to set the initial random weights of layers
	Activation	Relu and tanh	Activation function for the hidden layer
	Loss	Binary_crossentropy	The purpose of loss functions is to compute the quantity that a model should seek to minimize during training
	Optimizer	Adam	Adam optimization is a stochastic gradient descent method that is based on adaptive estimation of first-order and second-order moments

#### Classification

**FIGURE 3:**
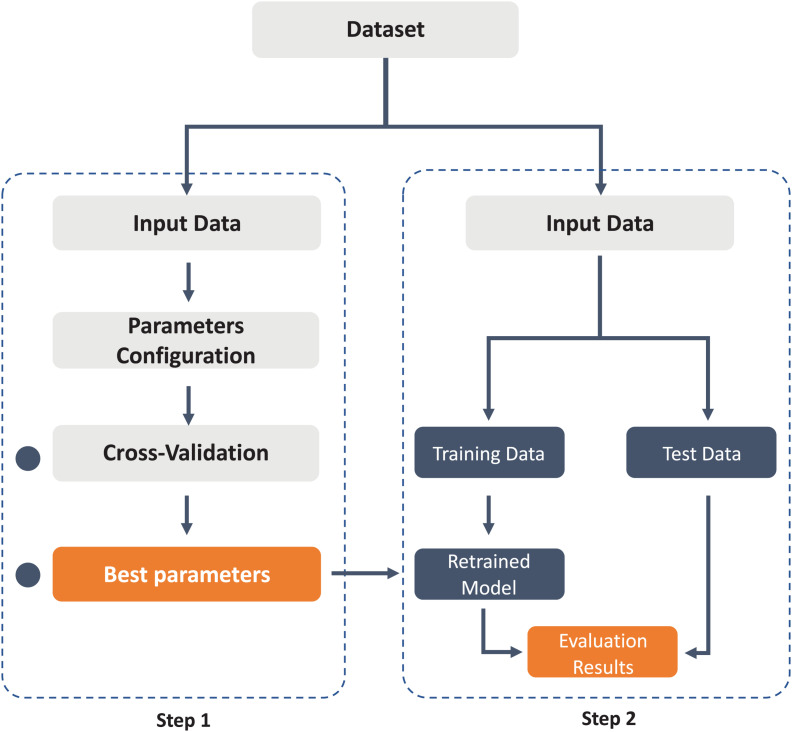
Workflow for cross-validation in model training and evaluation.

At this stage and taking into account the main insights obtained from the related work, machine learning classifiers are trained, evaluated, and validated through cross-validation and different precision metrics. Figure 3 describes the workflow adopted to validate the results of the classification models using cross-validation and to obtain a comparison of the best results in the model’s evaluation process.

All the data recorded from the trials that have been collected during the experimentation stage have been included in two different approaches, looking for the machine learning model with the best fit for the recognition and classification of emotions. The evaluations of users include negative samples (stimuli that the user qualified as unpleasant), neutral samples (periods when the user did not hear any stimuli), and positive samples (stimuli rated as pleasant by the user). Approach A includes negative (unpleasant) and neutral cases. Instead, approach B contains neutral and positive cases. The purpose of generating these approaches is to identify and recognize the difference between cases with different emotional state using machine learning models.

The percentage of samples for training and testing, the dimensionality of the feature vector, and the number of classes to evaluate the machine learning models’ performance are presented in Table 2. The features are extracted from each sensor’s EEG signal spectrum as a potential feature to feed the machine learning model.

**TABLE 2:** Dataset specification.

*Dataset (Approach)*	*Number of samples*			*Number of features (Features* = sensors * waves)	*Class*
			
	*Cross-validation*	*Training*	*Test*		
A1	100%	85%	15%	56 features = 14 sensors * 4 waves {delta, theta, alpha, and beta}	Unpleasant (-1) Neutral (0)
A2	100%	85%	15%	28 features = 14 sensors * 2 waves {alpha and beta}	Unpleasant (-1) Neutral (0)
B1	100%	85%	15%	56 features = 14 sensors * 4 waves {delta, theta, alpha, and beta}	Neutral (0) Pleasant (1)
B2	100%	85%	15%	28 features = 14 sensors * 2 waves {alpha and beta}	Neutral (0) Pleasant (1)

The number of features of each dataset depends on the type of waves included. Approaches A1 and B1 use 56 features for the binary classification of “unpleasant – neutral” and “neutral – pleasant,” respectively, extracted from 14 sensors (56 = 14 ^∗^ 4 {delta, theta, alpha, and beta}). Instead, approaches A2 and B2 employ 28 features for binary classification of “unpleasant – neutral” and “neutral – pleasant,” respectively, obtained from 14 sensors (28 = 14 ^∗^ 2 {alpha and beta}). In A2 and B2 approaches, it has been chosen to evaluate the alpha and beta frequencies since it is known from the literature that these frequencies reflect active mental states. Therefore, it is proposed to evaluate the importance of this type of frequencies in emotion classification tasks.

During classification tasks, first, the precision of each machine learning algorithm is validated using cross-validation (10 folds). Subsequently, each algorithm is evaluated by training a new model and with a set of samples reserved for its validation. The percentages of samples assigned in each step of the process have been described in Table 2. The results of the accuracy, precision, recall, and F1 score metrics will indicate the ability of the model to generalize new cases. Additionally, it is essential to validate that the model does not present a classification bias toward one of the problem classes.

## Results

In this section, the results of the proposed framework are presented. First, the percentage of samples evaluated as negative or positive per participant is reported. Later, machine learning algorithms for classifying affective states in people with visual disabilities are compared. Finally, each algorithm’s performance is analyzed, and precision analysis of the proposed models is presented.

### Pre-processing: Dataset Analysis

First, an analysis of each experiment’s responses reveals the percentage of samples that have been evaluated negatively or positively by all participants. Table 3 reports these results, ID Participant, Stimuli evaluated, % of negative samples, and % of positive samples associated with the evaluation by all participants.

**TABLE 3:** Percentages of samples negatives and positives evaluated by each participant.

*ID participant*	*Number of Stimuli evaluated*	*Negatives (%)*	*Positives (%)*
1	40	25.0%	75.0%
2	40	50.0%	50.0%
3	40	42.5%	57.5%
4	40	40.0%	60.0%
5	40	50.0%	50.0%
6	40	50.0%	50.0%
7	40	50.0%	50.0%

Once the EEG signals have been extracted and the average wave values processed from the 14 electrodes of the Emotiv Epoc+ headband, data analysis for inspecting, cleaning, and transforming data to highlight useful information has been performed. For visualizing this process, two boxplot diagrams have been defined. The usefulness of the boxplot diagram is that it offers, by simple visual inspection, a rough idea of the central tendency (through the median), dispersion (through the interquartile) of the symmetry of the distribution (through the symmetry of the graph), and possible outliers in each classifier. The rectangular part of the plot extends from the lower quartile to the upper quartile, covering the center half of each sample. The center lines within each box show the location of the sample medians. The whiskers extend from the box to the minimum and maximum values in each sample, except for any outside or far outside points, which will be plotted separately (Gonzalez-Carrasco et al., 2014; Molnar, 2019).

The detection of these outliers is crucial for understanding possible causes and implications of their presence (Cousineau and Chartier, 2010; Leys et al., 2013). Moreover, the importance of outliers has been studied in different domains and problems (Felt et al., 2017; Iwata et al., 2018; Peiffer and Armytage, 2019).

**FIGURE 4:**
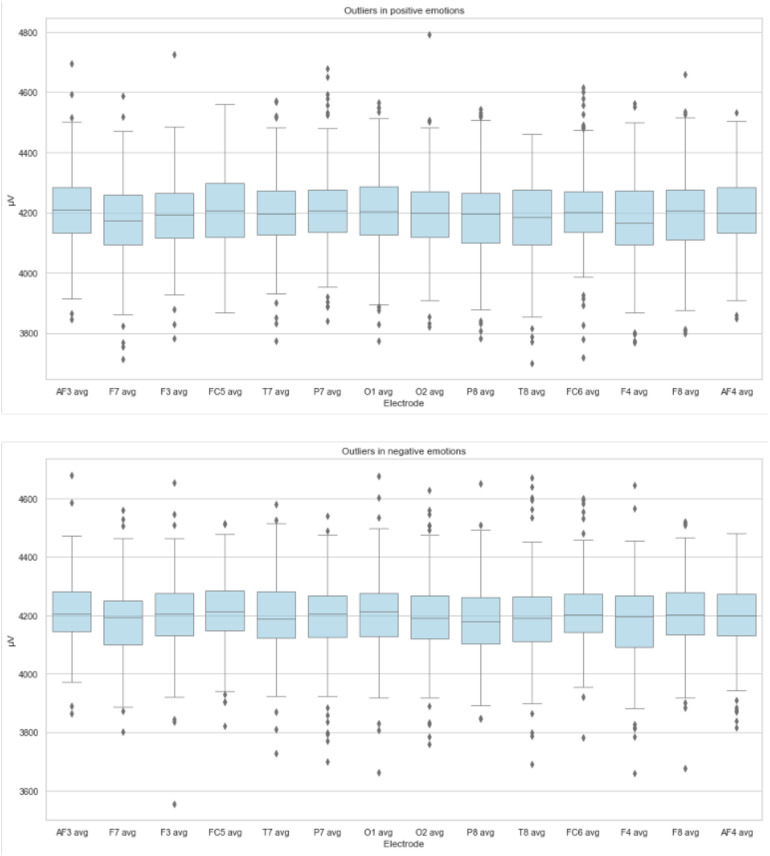
Variability of data and typical values in positive and negative emotions.

Figure 4 depicts the variability of the distribution of brain signal values in positive and negative emotions for the 14 electrodes of the BCI. Based on the visual analysis of the dataset, there are some outliers for the electrodes in the distribution of the values (median around 4200 μV). In the same way, the behavior of the signals is quite similar for positive and negative emotions. For this reason, it is essential to mention that the existence of outliers could determine the reaction and behavior of the brain to a given stimulus. Therefore, for the experimentation of this research, these outliers have been taken into account.

### Machine Learning

As mentioned above, the goal of the framework is to predict the basic emotions, positive or negative, of the participants. For this reason, at the machine learning stage, several machine learning algorithms and two approaches were evaluated to obtain the best performance. This section shows the process of evaluation and validation performed to determine the contribution of the research.

#### Classification

##### Approach A

**FIGURE 5:**
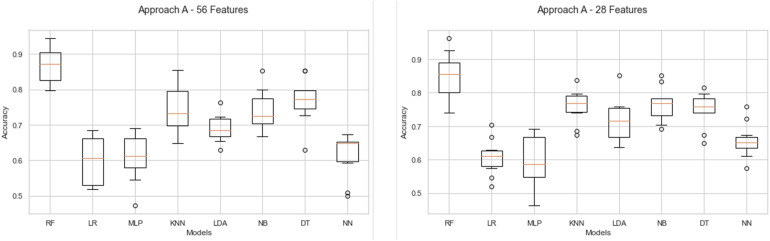
Behavior of classifiers in approach A1 and A2 with 56 and 28 features.

Firstly, the models’ performance for approaches A1 and A2 with 56 and 28 features, respectively, is presented in Figure 5. The configuration to evaluate the performance contemplates cross-validation with 10 folds for all models. Subsequently, the comparison of the performance of each model for approaches A1 and A2 is presented in Table 4. The first part describes each model’s results with the precision achieved during the cross-validation, the average, standard deviation, and the precision minimum and maximum. Next, the evaluation of the models is detailed; this includes the accuracy result for training and test tasks and the metrics obtained in precision, recall, and F1 score.

**TABLE 4:** Performance of classifiers for training and validation tasks in approach A.

Approach A																		

	A1 (56 features)										A2 (28 features)							
			
*Dataset*	*Cross-validation (10 folds)*																	

*Cross-validation (100%)*	Run	RF		LR	MLP	KNN	LDA	NB	DT	NN	RF	LR	MLP	KNN	LDA	NB	DT	NN
	**1**	0.87		0.56	0.69	0.80	0.76	0.80	0.78	0.67	0.82	0.60	0.60	0.84	0.73	0.69	0.78	0.67
	**2**	0.89		0.58	0.47	0.69	0.65	0.76	0.73	0.51	0.87	0.62	0.69	0.67	0.64	0.78	0.67	0.65
	**3**	0.82		0.67	0.55	0.85	0.67	0.75	0.76	0.65	0.84	0.55	0.55	0.75	0.67	0.78	0.78	0.65
	**4**	0.94		0.69	0.59	0.72	0.63	0.85	0.74	0.50	0.93	0.61	0.61	0.78	0.74	0.83	0.65	0.76
	**5**	0.80		0.63	0.57	0.65	0.67	0.67	0.80	0.65	0.89	0.63	0.56	0.80	0.70	0.78	0.74	0.65
	**6**	0.93		0.69	0.67	0.83	0.72	0.70	0.85	0.65	0.74	0.52	0.69	0.69	0.76	0.70	0.76	0.72
	**7**	0.91		0.52	0.61	0.72	0.69	0.78	0.63	0.67	0.80	0.70	0.46	0.74	0.85	0.72	0.81	0.65
	**8**	0.87		0.52	0.61	0.78	0.72	0.70	0.85	0.65	0.96	0.61	0.52	0.80	0.67	0.76	0.74	0.63
	**9**	0.80		0.52	0.65	0.74	0.69	0.70	0.76	0.61	0.89	0.67	0.69	0.76	0.76	0.85	0.80	0.61
	**10**	0.85		0.63	0.67	0.69	0.70	0.70	0.80	0.59	0.76	0.57	0.57	0.78	0.65	0.76	0.76	0.57
	**Avg**	***0.87***		***0.60***	***0.61***	***0.75***	***0.69***	***0.74***	***0.77***	***0.62***	***0.85***	***0.61***	***0.59***	***0.76***	***0.72***	***0.77***	***0.75***	***0.66***
	**Std**	***0.05***		***0.07***	***0.07***	***0.07***	***0.04***	***0.06***	***0.06***	***0.06***	***0.07***	***0.05***	***0.08***	***0.05***	***0.07***	***0.05***	***0.05***	***0.05***
	**Min**	***0.80***		***0.52***	***0.47***	***0.65***	***0.63***	***0.67***	***0.63***	***0.50***	***0.74***	***0.52***	***0.46***	***0.67***	***0.64***	***0.69***	***0.65***	***0.57***
	**Max**	***0.94***		***0.69***	***0.69***	***0.85***	***0.76***	***0.85***	***0.85***	***0.67***	***0.96***	***0.70***	***0.69***	***0.84***	***0.85***	***0.85***	***0.81***	***0.76***

	***Models evaluation***																	

***Training 85%***	**Acc**	**1.0**		**0.70**	**0.61**	**0.88**	**0.77**	**0.74**	**1.0**	**0.75**	**1.0**	**0.68**	**0.65**	**0.87**	**0.78**	**0.77**	**1.0**	**0.69**
***Validation 15%***	**Acc**	**0.83**		**0.56**	**0.55**	**0.70**	**0.72**	**0.70**	**0.72**	**0.63**	**0.85**	**0.51**	**0.61**	**0.67**	**0.78**	**0.77**	**0.74**	**0.60**
	***P***	−1	0.63	0.21	0.36	0.45	0.48	0.45	0.48	0.38	0.77	0.30	0.29	0.54	0.79	0.71	0.62	0.36
		0	0.94	0.71	0.90	0.86	0.84	0.84	0.84	0.80	0.90	0.62	0.64	0.73	0.78	0.79	0.83	0.65
	***R***	−1	0.86	0.23	0.86	0.68	0.59	0.64	0.59	0.55	0.83	0.28	0.07	0.48	0.52	0.59	0.72	0.17
		0	0.82	0.68	0.43	0.70	0.77	0.72	0.77	0.67	0.87	0.64	0.90	0.77	0.92	0.87	0.75	0.83
	***F1***	−1	**0.73**	**0.22**	**0.51**	**0.55**	**0.53**	**0.53**	**0.53**	**0.44**	**0.80**	**0.29**	**0.11**	**0.51**	**0.62**	**0.64**	**0.67**	**0.23**
		0	**0.87**	**0.69**	**0.58**	**0.77**	**0.80**	**0.77**	**0.80**	**0.73**	**0.88**	**0.63**	**0.75**	**0.75**	**0.84**	**0.83**	**0.79**	**0.73**

*Acc, accuracy; P, precision; R, recall; and F1, F1 score.*

*Bold and Italic values represent the highlight relevant values.*

In the results for approach A1 with 56 features, it is observed that RF is the model that best adapts to the problem of recognition of Negative and Neutral emotions. RF achieves a mean accuracy of 87%. Moreover, the performance of the KNN, NB, and DT models is very similar, reaching between 74 and 77%. Otherwise, the remaining LR, MLP, LDA, and NN models’ average performance is less than 70% mean accuracy. The evaluation of approach A1 with 56 features shows that the best model is RF with an average precision of 87% and a minimum accuracy of 80%, and a maximum of 94%. KNN behavior indicates an average of 75%, besides a minimum and maximum accuracy of 65 and 85%, respectively. On the other hand, NB averages 74% accuracy, with 67% as the minimum level and 85% as the maximum level. Meanwhile, the DT model achieves an average accuracy of 77%, with a minimum performance of 63% and a maximum of 85%. The average performance of the LR, MLP, LDA, and NN models is less than 70%, with minimal accuracy ranging from 44% up to a maximum accuracy of 76%.

Finally, the results of the validation of each model are shown. These indicate that RF achieves the best result for the classification of new cases; it obtains 83% accuracy. Additionally, the F1 score metric reports 73% for negative cases and 87% for neutral cases.

The performance of the models of approach A2 with 28 features (alpha and beta frequencies) is shown in the right part of Figure 5. The results again indicate that RF with 85% mean accuracy is the model with better levels. On the other hand, KNN, LDA, NB, and DT reach a mean accuracy between 70 and 80%. These models’ minimum values are between 64 and 69% and maximum values are from 81 to 85%. Finally, for LR, MLP, and NN models, the results indicate the lower performance with values less than 70% mean accuracy.

Likewise, in Table 4, the evaluation results of approach A2 with the alpha and beta frequencies are detailed. The results show that RF achieves the best mean result with 85% accuracy, with a minimum of 74% and a maximum of 96%. The results of the KNN model indicate a mean accuracy of 76% and a minimum and maximum of 67 and 84%, respectively. Instead, NB achieves a mean of 77%, a minimum of 69%, and a maximum of 85% accuracy. On the other hand, DT reports a mean of 75% accuracy and 69 and 85% as minimum and maximum, respectively. The LDA model achieves a mean of 72%, with a minimum of 64% and a maximum of 85%. The lowest performance models are LR, MLP, and NN; they show minimums of 46% to 57%, mean between 59 and 66%, and maximums of 69 up to 76% accuracy.

Finally, the models’ validation data with approach A2 with 28 features are presented in the lower part of Table 4. The data show that RF obtains the best result with 85% accuracy in the classification of new cases. Furthermore, this result is validated with the F1 Score metric, which shows 80% for negative classes and 88% for neutral classes.

Based on the results, it is observed that RF is the model that best adapts to the classification of new cases, considering the two scenarios proposed for approaches A1 and A2. Besides, the data show that RF is capable of classifying similarly for cases that are negative and neutral.

##### Approach B

**FIGURE 6:**
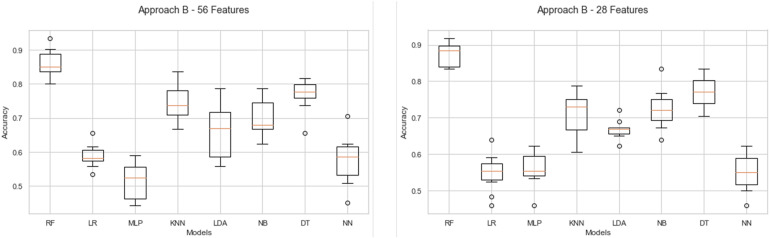
Behavior of classifiers in approach B1 and B2 with 56 and 28 features.

Initially, Figure 6 details the different models’ behavior evaluated for approaches B1 and B2 with 56 and 28 features, respectively. Next, the statistics of the behavior of the models on approach B are presented in Table 5.

**TABLE 5:** Performance of classifiers for training and validation tasks in approach B.

Approach B																			

	B1 (56 features)									B2 (28 features)									
				
*Dataset*	*Cross-validation (10 folds)*																		

*Cross-validation (100%)*	Run		RF		LR	MLP	KNN	LDA	NB	DT	NN	RF	LR	MLP	KNN	LDA	NB	DT	NN
	**c**		0.90		0.56	0.44	0.74	0.70	0.62	0.80	0.59	0.89	0.52	0.61	0.74	0.66	0.70	0.70	0.51
	**2**		0.90		0.57	0.46	0.70	0.72	0.79	0.66	0.62	0.89	0.56	0.54	0.66	0.67	0.74	0.82	0.62
	**3**		0.82		0.61	0.46	0.84	0.67	0.69	0.77	0.59	0.84	0.54	0.56	0.61	0.66	0.74	0.75	0.46
	**4**		0.84		0.66	0.48	0.79	0.74	0.64	0.80	0.52	0.92	0.57	0.56	0.75	0.69	0.70	0.70	0.54
	**5**		0.93		0.61	0.52	0.72	0.56	0.67	0.74	0.62	0.84	0.64	0.46	0.74	0.67	0.64	0.77	0.56
	**6**		0.85		0.59	0.52	0.74	0.79	0.75	0.79	0.70	0.89	0.46	0.62	0.72	0.67	0.67	0.80	0.54
	**7**		0.84		0.57	0.59	0.69	0.59	0.75	0.77	0.56	0.90	0.59	0.54	0.79	0.72	0.75	0.77	0.59
	**8**		0.85		0.57	0.56	0.79	0.57	0.72	0.75	0.51	0.92	0.57	0.62	0.75	0.62	0.69	0.80	0.61
	**9**		0.80		0.53	0.55	0.77	0.67	0.67	0.82	0.45	0.83	0.48	0.53	0.63	0.67	0.77	0.83	0.58
	**10**		0.85		0.62	0.58	0.67	0.58	0.67	0.78	0.58	0.85	0.55	0.55	0.70	0.65	0.83	0.73	0.50
	**Avg**		**0.86**		**0.59**	**0.52**	**0.74**	**0.66**	**0.70**	**0.77**	**0.58**	**0.87**	**0.55**	**0.56**	**0.71**	**0.67**	**0.72**	**0.77**	**0.55**
	**Std**		**0.04**		**0.03**	**0.05**	**0.05**	**0.08**	**0.05**	**0.05**	**0.07**	**0.03**	**0.05**	**0.05**	**0.06**	**0.03**	**0.05**	**0.05**	**0.05**
	**Min**		**0.80**		**0.53**	**0.44**	**0.67**	**0.56**	**0.62**	**0.66**	**0.45**	**0.83**	**0.46**	**0.46**	**0.61**	**0.62**	**0.64**	**0.70**	**0.46**
	**Max**		**0.93**		**0.66**	**0.59**	**0.84**	**0.79**	**0.79**	**0.82**	**0.70**	**0.92**	**0.64**	**0.62**	**0.79**	**0.72**	**0.83**	**0.83**	**0.62**

	***Models evaluation***																		

***Training 85%***	**Acc**	**1.0**			**0.67**	**0.55**	**0.86**	**0.69**	**0.70**	**1.0**	**0.75**	**1.0**	**0.61**	**0.58**	**0.81**	**0.69**	**0.74**	**1.0**	**0.62**
***Test 15%***	**Acc**	**0.88**			**0.62**	**0.52**	**0.79**	**0.71**	**0.77**	**0.79**	**0.52**	**0.88**	**0.60**	**0.57**	**0.74**	**0.66**	**0.73**	**0.80**	**0.60**
	***P***	-1		0.91	0.70	0.59	0.80	0.74	0.79	0.82	0.62	0.94	0.64	0.58	0.75	0.68	0.73	0.89	0.60
		0		0.84	0.51	0.35	0.77	0.65	0.74	0.74	0.41	0.81	0.52	0.25	0.72	0.63	0.72	0.72	0.57
	***R***	-1		0.89	0.66	0.70	0.88	0.80	0.86	0.84	0.54	0.85	0.72	0.94	0.83	0.81	0.85	0.76	0.94
		0		0.86	0.56	0.25	0.67	0.56	0.64	0.72	0.50	0.92	0.42	0.03	0.61	0.45	0.55	0.87	0.11
	***F1***	-1		**0.90**	**0.68**	**0.64**	**0.84**	**0.77**	**0.82**	**0.83**	**0.58**	**0.89**	**0.68**	**0.72**	**0.79**	**0.74**	**0.79**	**0.82**	**0.73**
		0		**0.85**	**0.53**	**0.29**	**0.72**	**0.60**	**0.69**	**0.73**	**0.45**	**0.86**	**0.46**	**0.05**	**0.66**	**0.52**	**0.63**	**0.79**	**0.18**

*Acc, accuracy; P, precision; R, recall; and F1, F1 score.*

*Bold and Italic values represent the highlight relevant values.*

Firstly, in the results of approach B1 with 56 features, it is observed that RF is the best model, achieving a mean precision of 86%. The models that reach similar values of 70% are KNN (74%), NB (70%), and DT (77%). In contrast, LR, MLP, LDA, and NN show a mean performance below 67% accuracy. The data RF shows a mean performance of 86% accuracy, and a minimum and maximum of 80 and 93%, respectively. On the other hand, KNN results indicate a maximum accuracy of 84%, a mean of 74%, and a minimum of 67%. NB model shows a mean accuracy of 70%, and minimum and maximum values of 62 and 79%. DT results indicate a mean accuracy of 77%, as well as a minimum and maximum of 66 and 82%, respectively. Finally, LR, MLP, LDA, and NN models show a mean accuracy of 66%, minimums from 45 to 56%, and maximums from 59 to 79%.

On the other hand, the models’ behavior for approach B2 with 28 features is represented in the right part of Figure 6. First, it is observed that RF obtains a mean accuracy of 87%. On the other hand, the KNN, NB, and DT models achieve a mean accuracy of more than 70%. Lastly, the LR, MLP, LDA, and NN models report results below 68%.

The performance statistics of approach B2 with 28 features show RF as the best model, which obtains a mean accuracy of 87%; maximum and minimum correspond to 93 and 83%, respectively. The KNN model achieves a mean accuracy of 71%, a minimum value of 65%, and a maximum of 79%. On the other hand, although NB and DT obtain a superior performance of 74% mean accuracy, it is below the RF model. The data minimum and maximum of NB are 64 and 83% accuracy. Instead, DT achieves a minimum value of 70% and a maximum value of 83%. The lowest results belong to LR, MLP, LDA, and NN models; they obtain a mean accuracy of less than 68%. The minimum percentages of these models range from 46 to 62% and the maximums range from 62 to 72%.

Finally, Table 5 presents the results of the validation of each model. The data indicate that RF is the model with the best result; it obtains 88% in the generalization of new cases, and F1 Score validation metric indicates 89% for neutral classes and 86% for positive classes.

According to the results for approaches B1 and B2, RF obtains the best performance in classifying neutral and positive cases. Besides, the data indicate that RF shows balanced performance in the classification of the proposed cases.

**FIGURE 7:**
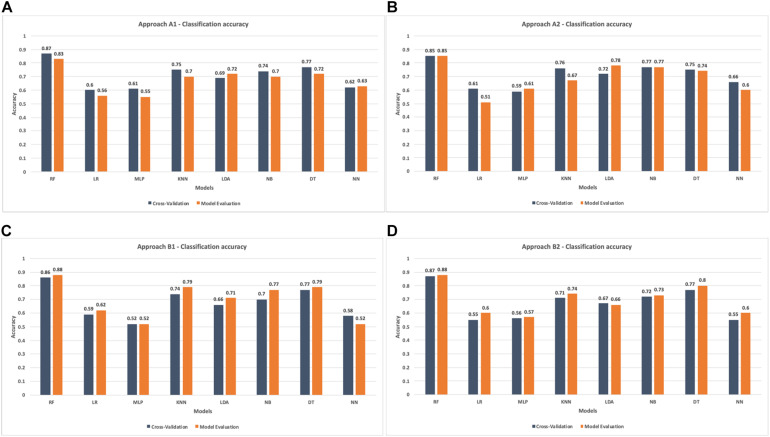
Accuracy comparison in cross-validation and models evaluation. **(A)** Classification accuracy in approach A-1. **(B)** Classification accuracy in approach A-2. **(C)** Classification accuracy in approach B-1. **(D)** Classification accuracy in approach B-2.

In order to demonstrate the best performance of the evaluated models, the average classification precision achieved in the cross-validation task and the result in the test task of each model are presented in the Figure 7. RF stands out for its uniform performance in the four evaluated approaches. Approach A1-56 obtains the best performance with 87% and 83% in the cross-validation and evaluation of the model, respectively. In comparison, approach A2-28 achieves the same result, 85% in both tasks. For approaches B1-56 and B2-28, RF achieves the best results. In B1-56, RF achieves 86% during cross-validation and 88% in model testing. In B2-28, RF obtains 87% and 88% accuracy in cross-validation and model evaluation, respectively.

**FIGURE 8:**
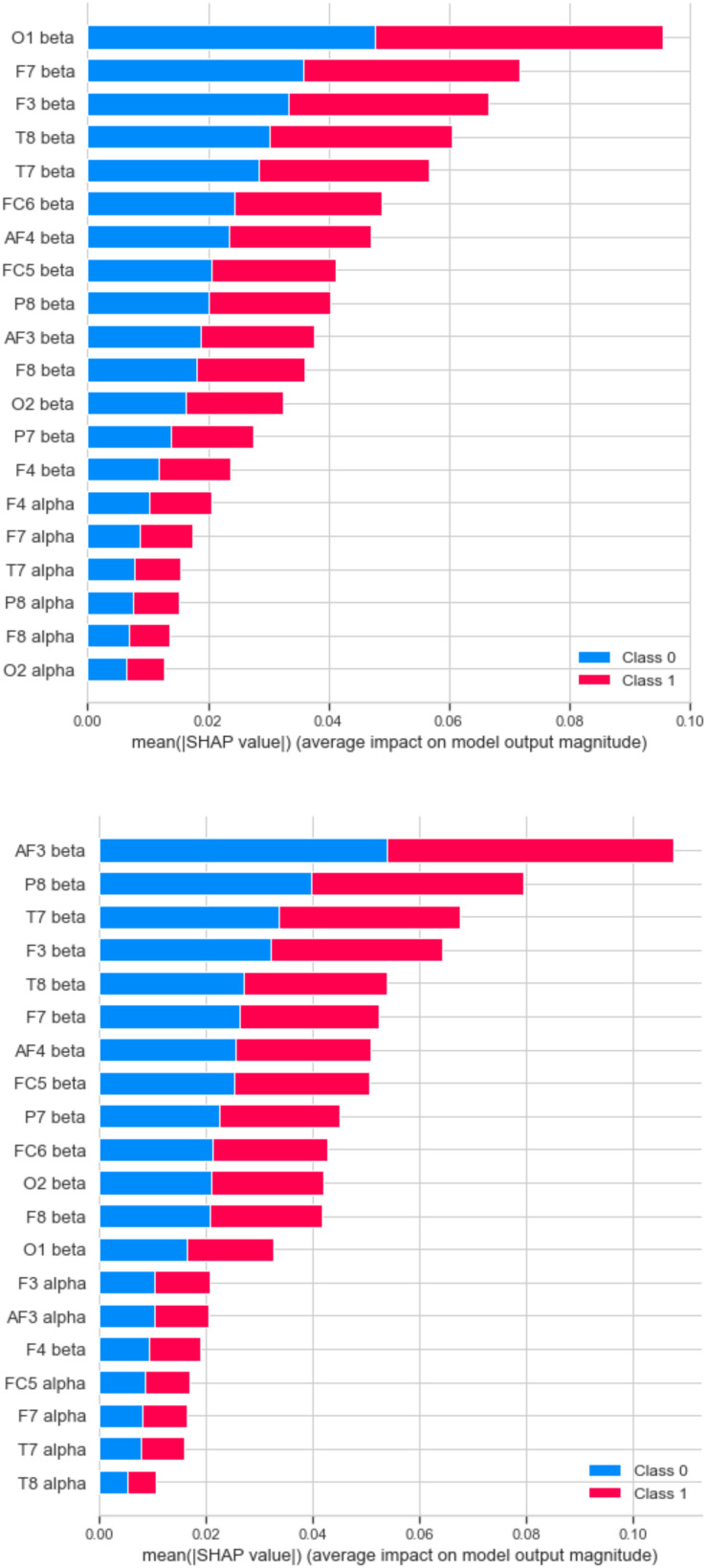
Feature sensibility analysis by SHAP technique. RF best result in approach A2 (upper) and B2 (lower).

The importance of the features of the A2 approach (28 features) and the B2 approach (28 features) is shown in Figure 8 [Tree SHAP technique (Lundberg et al., 2020)]. Feature relevance is calculated as the decrease in node impurity weighted by the probability of reaching that node. The node probability can be calculated by the number of samples that reach the node, divided by the total number of samples. In both cases, the higher the value, the more important the feature. Looking at the feature sensibility analysis, the relevant features are similar for both techniques. Therefore, future work should include significant features as a small set of electrodes (F3, T7, T8, F7, AF4, etc.) for trying to achieve similar performance with less complexity.

To evaluate the relevance of each participant’s dataset on the best model obtained, these are evaluated considering the results of the classification stage. Based on the data, RF is the model that is considered appropriate to evaluate all scenarios with the data per user. It is important to note that during data evaluation of each participant, a similar process is followed for the evaluation of each proposed approach, which consists of selecting the negative and neutral or neutral and positive samples, corresponding to approaches A1–A2 and B1–B2, respectively. The results per participant and the performance of the data on each model are presented in Table 6. It is identified that the performance of the data of participants 1, 2, 3, and 4 are similar, and they obtain accuracy results superior to the rest of the participants. Furthermore, it is possible to visualize that subjects 5, 6, and 7 obtain lower percentages of precision than the rest of the participants.

**TABLE 6:** Relevance analysis of data by participant.

*Approach/Participant*	*P1*	*P2*	*P3*	*P4*	*P5*	*P6*	*P7*
A1 (56 features)	1.00	0.99	0.99	0.99	0.45	0.45	0.45
A2 (28 features)	1.00	1.00	0.98	0.98	0.52	0.5	0.45
B1 (56 features)	1.00	0.99	0.99	0.98	0.45	0.5	0.5
B2 (28 features)	0.99	1.00	0.98	1.00	0.55	0.5	0.57
**Avg**	**0.99**	**0.99**	**0.98**	**0.98**	**0.49**	**0.48**	**0.49**

On the other hand, it is observed that the A2 and B2 models that include 28 features with the alpha and beta frequencies obtain slightly higher results compared to the models with 56 features that include the alpha, beta, theta, and delta frequencies.

Finally, Table 7 includes an accuracy comparison among relevant related research and the approach proposed by the authors (at the bottom of the table).

**TABLE 7:** Comparison of results with other related approaches.

Classifier	Accuracy (%)	Comment
SVM (Patil and Behele, 2018)	91.96	Multiclass SVM is used for the multiple classification of four different emotions
SVM (Ramirez and Vamvakousis, 2012)	86.33	Machine learning techniques work well and are generally able to distinguish patterns to classify a person’s emotional states
SVM (Ackermann et al., 2016)	52	SVM is a robust algorithm when it has few EEG signal characteristics, and it also has the ability to classify a person’s emotions
MLP (Sánchez-Reolid et al., 2018)	96	It is mentioned that an ANN based on a multilayer perceptron (PMC) is a competent approach to classify emotions
KNN (Mehmood and Lee, 2015)	61	Indicates that the KNN algorithm for the classification of emotions will have better results than SVM
KNN (Kaundanya et al., 2015)	100	It is identified that the smaller the number of neighbors, the results obtained in the identification of emotions are better
RF	85 Negatives 88 Positives	In this research, RF is a useful algorithm for the classification of emotions, from alpha and beta brain signals
DT	74 Negatives 80 Positives	In this research, DT is an algorithm that has a non-uniform behavior for the classification of negative and positive emotions

## Discussion

This section discusses the main insights and breakthroughs regarding the results obtained with the framework proposed in this work.

Firstly, this study presents a new scenario for the recognition and classification of emotions in people with visual disabilities, a group of people not previously evaluated. The work defines and implements a framework through a non-invasive BCI (Emotiv Epoc+) with a set of auditory stimuli. From the records obtained, two datasets were formed with the stimuli classified as negatives-neutrals and neutrals-positives. Subsequently, the model’s RF, LR, MLP, KNN, LDA, NB, DT, and NN were configured and evaluated to identify the model with the best performance in recognition and classification tasks emotions from EEG data.

The results show that in the individual evaluation of the stimuli, participants 1, 3, and 4 evaluated more than 50% of the stimuli positively. Instead, participants 2, 5, 6, and 7 had a balanced evaluation toward stimuli. On the other hand, it is possible to observe brain signal variability concerning stimuli considered positively and negatively (see Figure 4). Although the behavior shows similar values of 4,200 μV, outliers were recorded in the data. That could indicate a different reaction of the participants to the presented stimuli. It is important to note that the entire data have been considered during the machine learning tasks. That is, the outliers have not been omitted. Additionally, the data show that RF is the model with the best performance during classification tasks. In evaluating results, the RF model in the A1-56 and A2-28 approaches to negative emotions achieved a classification accuracy of 83% and 85%, respectively. In turn, with the positive emotions of approaches B1-56 and B2-28, he obtained an accuracy of 88% in both cases. The best results obtained from the RF model in approaches A2-28 and B2-28 with negative and positive emotions and analysis of the features’ importance allowed us to recognize that the beta frequencies related to the frontotemporal areas of the brain are important in the decision making of the models. On the other hand, the results show that the algorithms LR, MLP, KNN, LDA, NB, DT, and NN obtain a lower performance compared to RF (see Figure 7). Although, in the validation of the models, it is observed that DT and KNN obtain acceptable results for the classification of positive emotions, this result is not consistent with the identification of negative emotions. Therefore, these models tend to classify toward one type of emotions.

The analysis of the participants’ brain signals’ dataset allows identifying the variability in each subject’s data. This characteristic is relevant because it is considered relative to the perception of each subject toward each stimulus. This agrees with what is stated by Anagnostopoulos et al. (2012), which mentions that people’s emotional perception commonly differs. The evaluation of the different machine learning models and, according to the results obtained from the RF algorithm, their performance coincides with the findings reported in Ackermann et al. (2016). It is stated that RF is a robust algorithm in the processing and recognition of patterns from EEG signals. The test’s precision indicates that RF is an algorithm useful for classifying emotions using EEG signals. Moreover, in the validation of the RF model, it achieves the best results with an accuracy of 85% for negative emotions and 88% for positive emotions. Therefore, the RF classifier shows that it learns in both classes.

Considering the types of frequencies, delta, theta, alpha, and beta, different machine learning models have been trained and evaluated to determine their ability to recognize and classify different affective states of a group of people with visual disabilities. The data show that models that consider alpha and beta frequencies perform slightly better than models that consider all frequencies. The results show that models that consider alpha and beta frequencies perform slightly better than models that consider all frequencies. These results coincide with Ramirez and Vamvakousis (2012), who mention that the most important frequencies are alpha waves (8–12 Hz), which predominate in mental states of relaxation, and beta waves (12–30 Hz), which are active during states with intense mental activity. Similarly, the results show that the frontotemporal brain areas associated with the beta frequency show the greatest contribution to the performance of the models (see Figure 8). Finally, the model’s performance proposed in this research reaches values comparable to other research (Fdez et al., 2021) to classify emotions into two categories (negative and positive).

Due to the limitations of this study in processing brain activity from EGG data, it is important to consider different signal acquisition and processing aspects. The first factor needs to check all the sensors; this avoids errors in the recorded data. On the other hand, signal processing includes considering aspects such as noise generation. Previous tests are necessary to minimize noise generation, ensuring that the participant feels comfortable with the device; this avoids unexpected data generated by involuntary movements.

Finally, one way to respond to the study limitations is to increase the participant population in the experimentation stage. This would result in a greater number of evaluations toward the stimuli. Another criterion is to expand the number of stimuli and experimental sessions to obtain a large amount of information related to people’s emotional perception of different auditory stimuli. In turn, this would allow extending the analysis of the behavior of brain signals and their response to specific stimulus.

As future work, the authors propose to extend the framework with more techniques such as recurrent neural networks (RNN), convolutional neural networks (CNN), or long short-term memory (LSTM). Those techniques could be compared with the current ones for obtaining a more in-depth study of brain waves and emotions in people with visual disability. Additionally, it is proposed to explore the brain regions’ behavior using 2D and 3D maps of the participants’ brain activity. This process will allow recognizing the brain areas that reflect high or low activity during the stimulation process. Future research can also consider incorporating data from other sources; i.e., the framework will have more than one entry at the same time. Adding data from a new source, other than EEG brain signals, will provide more knowledge for the classification of a person’s affective state and could improve the accuracy of the model. Also, as exposed in the section “Discussion,” a reduced dataset can be tested, taking into account the relevant features of the sensitivity analysis. Besides, as stated in the related work, many of the models proposed for the classification of emotions have not been evaluated in real time. Therefore, the authors take into account the assumptions made by Lotte et al. (2018) and propose as future research to adapt and evaluate the framework as a BCI for real-time emotion recognition.

It is remarkable that, although the participants of this research have visual disabilities (population not previously tested), the authors’ proposal reached similar levels of accuracy compared to other research for the classification of people’s emotions.

## Conclusion

It should be taken into account that emotions play an essential role in many aspects of our daily lives, including decision making, perception, learning, rational thinking, and actions. Likewise, it should be considered that the study of emotion recognition is indispensable (Pham and Tran, 2012).

In this work, the authors have explored and analyzed a previously unreported scenario, the classification of emotions in people with visual disabilities. The most important aspects of the framework are as follows: (i) It is a twofold framework. The first is mainly focused on data acquisition (signal extraction) with a BCI device using auditory stimuli. The second is concerned with analysis techniques for the modeling of emotions and machine learning models to classify emotions. (ii) The framework can be expanded with more machine learning algorithms, and therefore it increases the flexibility. (iii) Experimentation is focused on people with visual disabilities. Experimentation results show that 28 feature approaches, including alpha and beta frequencies, performed best for emotion recognition and classification. According to these models’ performance, the achieved accuracy is 85 and 88% in the classification of negative and positive emotions, respectively. Therefore, it is considered that feature selection plays a key role in classification performance. Also, an analysis of features illustrates that the brain’s frontotemporal areas linked to beta frequency have the most significant contribution to the proposed models’ performance. Finally, it has been proposed to continue research based on brain signals and to incorporate new sources of information from people with disabilities, to develop new ways of communication and technological interaction that will allow them to integrate into today’s society.

## Data Availability Statement

The datasets presented in this article are not readily available because data protection. Requests to access the datasets should be directed to corresponding author.

## Ethics Statement

Ethical review and approval was not required for the study on human participants in accordance with the Local Legislation and Institutional Requirements. The patients/participants provided their written informed consent to participate in this study.

## Author Contributions

JL-H, IG-C, and JL-C: writing—original draft preparation. JL-H, IG-C, JL-C, and BR-M: writing—review and editing. IG-C and BR-M: supervision and funding acquisition. All authors contributed to the article and approved the submitted version.

## Conflict of Interest

The authors declare that the research was conducted in the absence of any commercial or financial relationships that could be construed as a potential conflict of interest.
